# Social participation and territory: possible dialogues for the sustainable management of cultural heritage

**DOI:** 10.1590/S0104-59702023000100070

**Published:** 2023-12-15

**Authors:** Marcos José de Araújo Pinheiro, Roberta dos Santos de Almeida

**Affiliations:** i Professor, Pós-graduação em Preservação e Gestão do Patrimônio Cultural das Ciências e da Saúde , Casa de Oswaldo Cruz / Fiocruz . Rio de Janeiro – RJ – Brasil . marcos.pinheiro@fiocruz.br; ii Bolsista em pesquisa, Casa de Oswaldo Cruz / Fiocruz . Rio de Janeiro – RJ – Brasil . arq.robertasantos@gmail.com

**Keywords:** Social participation, Cultural heritage, Territory, Integrated conservation, Sustainability, Participação social, Patrimônio cultural, Território, Conservação integrada, Sustentabilidade

## Abstract

Based on the concepts of culture, territory, integrated conservation, and sustainability, this article analyses the meaning of social participation in the recognition, appropriation, preservation, and enhancement of cultural heritage. The object of analysis is the Manguinhos Historical Architectural Site, at Fundação Oswaldo Cruz, and its Requalification Plan, which aims to transform it into a “park campus.” A diagnostic study was conducted of social participation in this heritage, which found that greater participation should be encouraged if the complex is to be consolidated as science and health heritage and also appropriated by the territory as an asset of symbolic, cognitive, and identity values and a structuring element for sustainable development.

## Resumo

A partir dos conceitos de cultura, território, conservação integrada e sustentabilidade, o artigo analisa o significado da participação social em processos de reconhecimento, apropriação, preservação e valorização do patrimônio cultural. Adota como objeto o Núcleo Arquitetônico Histórico de Manguinhos, da Fundação Oswaldo Cruz, e seu Plano de Requalificação, que visa transformá-lo num “ *campus* parque”. Tem base num diagnóstico sobre a participação social, que aponta a necessidade de sua ampliação para a consolidação desse núcleo como um patrimônio cultural não só para as ciências e a saúde, mas também por sua apropriação pelo território como um bem de valores simbólico, cognitivo e identitário e como elemento estruturante ao desenvolvimento sustentável.

The concept of territory is not restricted to the sense of a delimited geographic space with specific physical and structural features. It is a multifaceted, complex network involving subjects, objects, relationships, and diverse interactions, creating a socially constructed polysemic space ( Santos, 2017 ). This notion is also linked to the exercise and dispute of power in the political, economic, and social arenas over a certain area. That is, in order to address a territory, it is important to understand the multiple things that integrate with the (individual, social, or collective) actors that are manifested in that space.

According to Haesbaert (2004) , territories are immersed in relations of domination and/or appropriation of society and space, whose physical structures may be manipulated and appropriated by different subjects, forging relationships and attributing meanings and values, and expressing different cultural manifestations. While the perspective of domination is centered on the dynamics of capitalist production and accumulation, the perspective of appropriation is linked to the cultural and symbolic meaning of life.

The Oswaldo Cruz Foundation (Fundação Oswaldo Cruz, Fiocruz) is a public institution of great international importance in the area of science and health, which operates under the auspices of the Brazilian Ministry of Health. It is headquartered in Manguinhos, a district in Rio de Janeiro. The site was chosen originally with an eye to the development of a maritime and rail transport system in the late nineteenth century, resulting from the expansion and transformation of the urban fabric and the shift from rural to industrial activity. However, nowadays it represents yet another example of segregated urban development, whose deepening socioeconomic divides are exemplified in the *favela* territories of Rio de Janeiro. In response to this scenario, Fiocruz has accumulated a history of action in the territory for the promotion of health and reduction of socioenvironmental inequities. The paucity of adequate infrastructure and services needed to ensure a decent quality of life for the local people, including the social factors that affect their health conditions, is the justification and driving force for the institution’s actions geared towards reducing inequalities on a territorial scale.

Since 2003, Fiocruz has had a department linked to its President’s Office dedicated to coordinating actions and developing communication networks and direct relationships, called the Social Cooperation Coordination. Since its inception, this unit has consolidated a number of instruments, policies, and actions for the territory. Linked to this unit are the Fiocruz Strategies for Agenda 2030 (EFA, 2030), instituted as of 2017 with the aspiration to achieve the five “Ps” of sustainable development set forth by the United Nations ( ONU Brasil, 2023 ): people, planet, prosperity, peace, and partnership. Their main goal is to analyze and prospect strategic actions for health, development, and sustainability on an institutional level in the medium and long term.

Another important aspect of Fiocruz’s work is the Institutional Program for Sustainable and Healthy Territories (Fiocruz, 2019), which takes the combined perspectives of sustainability and territory to support and articulate institutional knowledge and experiences, featuring the engagement of social groups present in socially and environmentally vulnerable territories, constituting a collaborative network capable of consolidating sustainable practices and public policies related to the subject. The program seeks to align its agenda with different initiatives and institutional programs, such as the Fiocruz Strategies for Agenda 2030 itself and the Social Cooperation Coordination’s actions, interfacing with national strategies and policies that target territorial enhancement.

The Fiocruz headquarters occupy what was once farmland – a single plot in Manguinhos – whose boundaries have undergone successive changes as land has been reclaimed, rivers rerouted, and access roads built. In the 1940s, the land occupied by the institution was severed in two by the Brasil Avenue highway, since which time its internal community of workers has come to understand it as two separate areas. The Manguinhos Campus is the more extensive area, located on the west side of Brasil Avenue and bordering the Manguinhos neighborhood. It contains a large green area, an area of historical preservation, and a set of buildings to house the different institutional units and departments. On the east of the highway is the Expansion area, which borders the Maré neighborhood. It was first occupied in the 1970s during the Castello Branco government, when the building for the Health Precinct, an arm of the Ministry of Health, was opened (Oliveira, Costa, Pessoa, 2003, p.158). The land and the building were reappropriated by Fiocruz in the 1980s and renamed the Expansion of the Manguinhos Campus. This area now harbors a variety of activities under different Fiocruz institutes.

**Figure 1:**
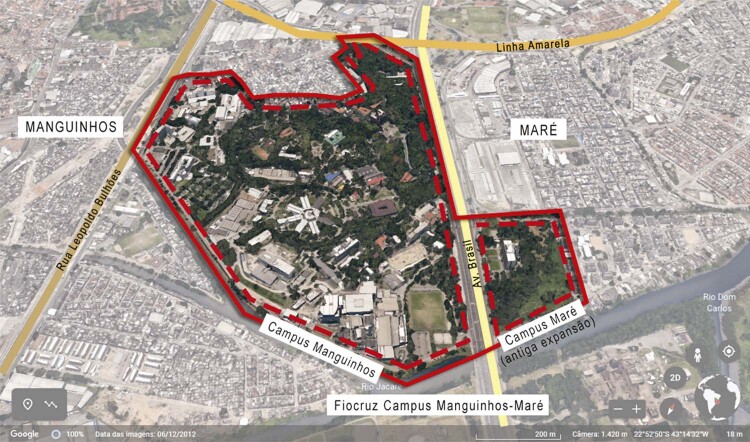
: Bird’s eye view of the Fiocruz Manguinhos-Maré Campus (adapted from Google Earth 2023)

In 2022, a new nomenclature was adopted for the whole Fiocruz headquarters: Fiocruz Manguinhos-Maré Campus. The objective is to counter the idea of division associated with the territory and to challenge the idea of the newer area as an appendage or expansion of the Manguinhos Campus; an occupation and not an independent campus in its own right. With this new name, the former expansion area is officially incorporated into the boundaries of Fiocruz, even if it is situated in a different neighborhood and plot of land from the Manguinhos Campus and on the opposite side of Brasil Avenue (see Figure 1 ).

This renaming has helped, symbolically, to overcome the physical barrier between the two campuses and the surrounding communities. Indeed, the Brasil Avenue, despite having been conceived as a strategic arterial road connecting the city to surrounding areas, has had the paradoxical effect of interfering with and dividing areas constituted as socio-environmental spaces before its construction. The renaming also corroborates the Fiocruz policy of recognition and induction in favor of the territoriality already manifested in different strategies pursued by its different units and bodies, making Fiocruz an inseparable part of the territories of Manguinhos and Maré.

In an urban context, the Fiocruz territory can be seen to share a history of socioenvironmental transformations. In particular, it is intertwined with the origins and occupation of one of the *favelas* in the Manguinhos neighborhood called Morro do Amorim, which developed in response to the construction and operation of Fiocruz in the early decades of the twentieth century.^1^ Therefore, understanding the Fiocruz Manguinhos-Maré Campus means legitimizing a longstanding relationship between the institution and the sociocultural groups and communities in these territories.

**Figura 2:**
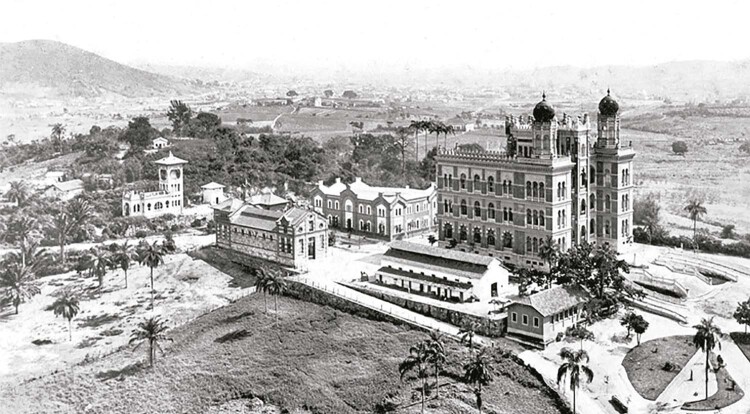
: Archive photograph of the Manguinhos Historical Architectural Site, undated (Departamento de Patrimônio Histórico/Casa de Oswaldo Cruz/Fiocruz)

The main focus of this study – the Manguinhos Historical Architectural Site (Núcleo Arquitetônico Histórico de Manguinhos, Nahm) – is part of this context and is articulated with these territorial dynamics. The complex is a historical site composed of a group of buildings and gardens conceived by Oswaldo Cruz during his administration in the early days of the institution’s trajectory. Built between 1904 and 1922, it was designed by the Portuguese architect Luiz Moraes Jr., who endowed the main building with decorative elements characteristic of neo-Moorish and eclectic architecture. The complex contains the Moorish Pavilion (Pavilhão Mourisco) (1905-1918), the Plague Pavilion (Pavilhão da Peste) (1904-1905), the Stables (Cavalariça) (1904), the Figueiredo Vasconcelos Pavilion, also known as Quinino (1919-1921), the Tea House (Casa de Chá) (c.1905) and its annex (c.1920), the Dovecote (Pombal) (1904), as well as the green areas and gardens of Pasteur Square (Praça Pasteur) and Oswaldo Cruz Path (Caminho Oswaldo Cruz) ( Figure 2 ).

Alongside these historical buildings, the campus’s cultural heritage also consists of the Modern Complex, whose buildings, inspired by the ideals of modern architecture, were constructed in the 1940s and 1950s.^2^ The value of this heritage transcends its architecture, extending to the history of science and health in Brazil, constituting a visual and symbolic representative of a Brazilian institution whose most recent feat in the world scenario was its important role in overcoming the public health crisis sparked by the coronavirus SARS-CoV-2. The value of the Manguinhos Campus is recognized by actors from civil society and the state; its integrity is protected by municipal, state, and national institutions; and it is a notable landmark in the urban landscape of Rio de Janeiro, since the Moorish Pavilion, occupying land at a higher elevation than the surrounding area, stands out against the skyline for its striking architecture.

As an integral part of the trajectory of Fiocruz, the Nahm is endowed with the accumulated experiences and dynamics of different individual and collective actors from society, forging emotional ties and participating in the attribution of multiple meanings and values. Taken together, these elements confirm the territoriality of this heritage and its inseparability from the territories of Manguinhos and Maré.

Currently, the administrative activities and research laboratories housed at the Nahm coexist with its other functions as a feature of the tours arranged for the public by the Fiocruz Museum of Life (Museu da Vida Fiocruz) and as a venue for public exhibitions. The museum, subordinated to Casa de Oswaldo Cruz (COC),^3^ has the role of promoting and popularizing science and research through cultural and educational actions. Together with the Department of Historical Heritage (Departamento de Patrimônio Histórico, DPH/COC),^4^ it is a social actor that mediates this cultural heritage, providing different experiences in these spaces, which help create memories and engage actors from the territory with the cultural heritage.

In the 2000s, Fiocruz was asked by the federal heritage protection agency, Institute of National Historical and Artistic Heritage (Instituto do Patrimônio Histórico e Artístico Nacional, Iphan), which had previously listed the heritage of the Manguinhos Campus, to draw up a master plan specifically for that heritage,^5^ since the existing master plans^6^ did not take account of the urban and architectural transformations that had occurred in the interim. In response, DPH/COC was put in charge of developing the plan for the occupation of the listed area of the Fiocruz Manguinhos Campus (Occupation Plan). Produced in 2011,^7^ the plan included a proposal to consolidate the vocation of this urban space as a “park campus,” understanding it as a healthy, safe, comfortable, and culturally enriching environment for its employees and visitors ( Fiocruz/COC, 2011 ). The Occupation Plan provided the basis for a strategic plan for the Nahm that combined a number of political and economic advantages with the preservation and promotion of this cultural heritage. The institution’s normative framework, which includes urban and heritage management policies, embodies considerable accumulated experience from a democratic and sustainability perspective.^8^ Taken together with the historical institutional values and principles, it was the *leitmotiv* that guided and enabled the preparation of the Nahm Requalification Plan in 2014.

**Figure 3:**
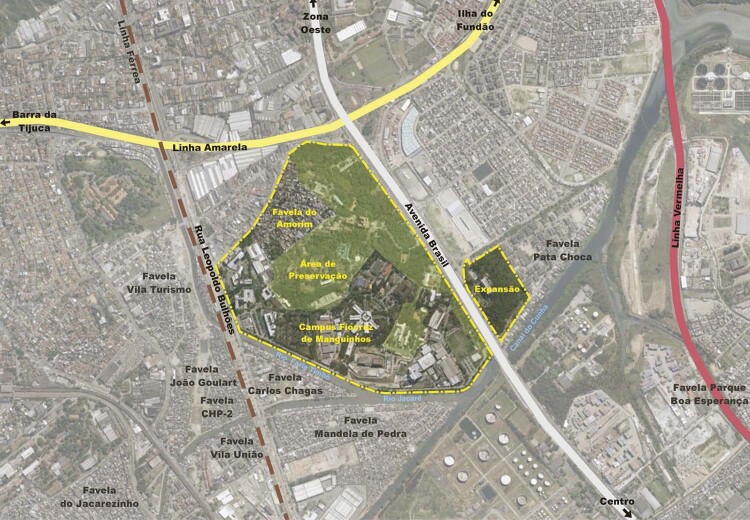
: Map showing the Manguinhos Campus Preservation Area ( Fiocruz/COC, 2011 , p.71)

**Figure 4:**
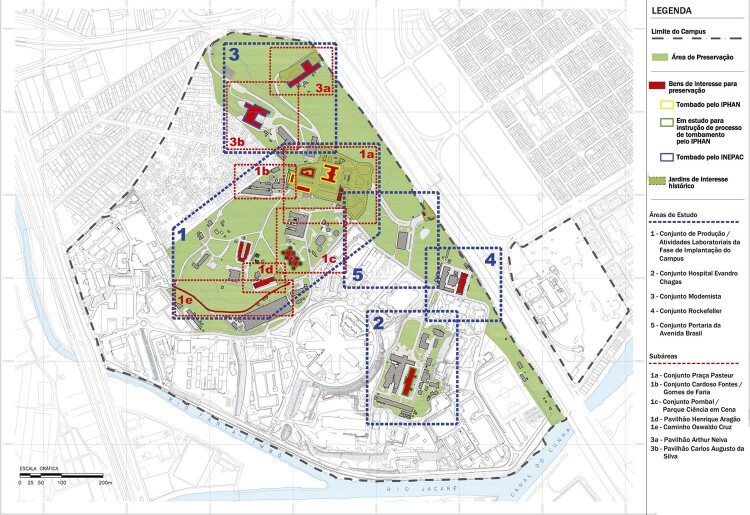
: Map showing the research areas of the Occupation Plan for the Manguinhos Campus Preservation Area ( Fiocruz/COC, 2011 , anexo 3, mapa 1: Áreas de estudo)

This brings us to the core of this article, namely, an analysis of the social aspects of the Nahm Requalification Plan, adopting social participation as an approach for its ability to take account of the different actors that constitute the Manguinhos-Maré territory and to contribute to its sustainable development.

## Manguinhos Historical Architectural Site Requalification Plan and its social relevance

In order to address the social dimension of cultural heritage, taking it as a set of things, relationships, and meanings attributed by different actors and groups in society over time, it is necessary to work with an expanded conception of culture and the notions of integrated conservation and sustainability. These concepts converge in the idea of the inseparability of cultural heritage from social participation. This in turn stems from the fact that the value of a cultural asset is not something innate or inherent to it, but an attribute lent it by a population in a certain context, place, and time, as well as the idea that its social appropriation occurs in direct proportion to the participation of society in its recognition as heritage.

In the Requalification Plan, health and culture are understood as follows. Health is understood in its expanded conception as having have economic, political, social, and cultural facets, while culture – and therefore cultural heritage – is understood from the prevailing anthropological perspective to transcend the ideas of high, low, or mass culture. This conception understands the actions and output resulting from the human quest for meanings and achievements as culture, associating it with “behavioral, spiritual, and material products of human social life as defined in the nineteenth century by Edward Tylor” (Pinheiro, Nascimento Jr., 2020, p.651). Similarly, as set forth by the United Nations Educational, Scientific and Cultural Organization Convention on the Protection and Promotion of the Diversity of Cultural Expressions (Unesco, 2007), of 2005, it is marked by diversity, which is seen as a cornerstone of identity, development, and coexistence between peoples and nations.

As for sustainability, considering the various discussions about this subject conducted in global forums, mainly since the 1972 United Nations Conference on the Human Environment in Stockholm, it can be understood as an ongoing social construct, the result of repeated debates over time that have consolidated principles and good practices in order to meet the needs and aspirations of present and future generations. Its meaning is rooted in the environmental, social, and economic dimensions,^9^ and is supported by other concepts specific to each dimension, having been incorporated extensively into political agendas through the 2030 Agenda.

When applied to heritage, the concept of sustainability can refer to values and identities developed over time ( Acselrad, 1999 ). According to UN Sustainable Development Goal (SDG) 11,^10^ providing for the protection and safeguard of cultural heritage directly influences people’s quality of life, making it one of the steps towards making “cities and human settlements inclusive, safe, resilient and sustainable” ( ONU Brasil, 2023 ). This perspective is in line with the broader understanding of health that guides the work of Fiocruz, whereby various social, environmental, cultural, economic, ethnic/racial, psychological, and behavioral variables are understood to affect people’s daily life, their well-being, and, therefore their health, as encapsulated in the idea of the socioenvironmental determinants of health (Buss, Pellegrini Filho, 2007).

For its part, the concept of integrated conservation understands heritage in conjunction with the urban fabric as being shaped by historical and social processes. It demands shared responsibility between local authorities and the population to constitute, preserve, and manage the heritage, highlighting heritage education as a means to raise the necessary awareness and spread and update knowledge, on the understanding that “a policy of conservation also means the integration of the architectural heritage into social life” ( Conselho da Europa, 1975 , p.6).

Reflections on the sustainable management of cultural heritage must therefore focus on its (inter)relations with the various actors that make up society, seeing them as being capable of appropriating, caring for, transmitting, and resignifying the heritage. This means there is an important point of contact between the concepts of heritage, integrated conservation, and sustainability: social participation.

In theory, social participation is the essence of democracy, based on the redistribution of power and the expansion of public debate on decisions that affect a society and its different population groups. The involvement of citizens in political processes always spawns a degree of dispute, which occurs on different, more or less legitimate and effective levels. Arnstein (1969) lists eight categories (“rungs on a ladder”), which she subdivides into three, to classify degree of social participation according to the power that citizens are able to exercise in political processes.

The bottom two categories, or rungs, are classified as non-participation, and the next three are called “tokenism,”^11^ signifying participation that is only figurative, symbolic, or manipulative, without sufficient scope to effect transformation, leaving power structures intact, concentrated in the hands of managers or top decision-makers. In contrast, the three rungs at the top of the ladder represent the highest levels of social participation, in which citizens are able to exercise the power of speech, listening, deliberation, negotiation, and decision-making in shared responsibility with managers.

When it comes to policies for the preservation of cultural heritage, participatory processes can engage and empower citizens from the constitution of the heritage itself, its relationships, and the attribution of values and meanings, to decision-making for its shared management.^12^ They also include the integration of heritage in the social life of citizens, that is, in the degree to which they appropriate and use this heritage.

Following the Lisbon Charter (Carta de Lisboa, 1995), we can consider requalification as a management strategy that aims to review some activity, adapting it to the place and context in which it occurs. It must provide for social participation, integrating all the stakeholders, who in turn must know the heritage and the process. It can also be understood as a heritage valuation strategy, oriented towards the recognition of the values of the heritage and its resonance in society ( Choay, 2017 ; originally published in 1925).

In its reference document, the Nahm Requalification Plan advocates the principles of sustainability and integrated conservation, which makes it strategic for deepening the relationship between society and its cultural heritage, and for achieving a democratic and participative process. It differs from architectural requalification, because of its scope, and also from urban requalification because

it is not about revitalizing or transforming historical areas and buildings that are little used or unused by a centenary institution into a museological space, as occurs in many interventions of this nature, but about reconciling its institutional and scientific breadth and vigor, its identity and its ethos, with its inherent vocation as a public space that is also dedicated to scientific education and the daily reality of populations, notably those in a vulnerable state (Almeida, Pinheiro, 2020, p.13).

The first step in the creation of the Nahm Requalification Plan was the approval of the Occupation Plan for Manguinhos Campus Preservation Area. This was enabled by a favorable institutional environment marked by a top management team that was sensitive to cultural heritage and science education, as well as the possibility of erecting new buildings in the Manguinhos and other campuses in Rio de Janeiro. This meant the administrative activities in the Nahm could be gradually substituted by others that enhanced and preserved its historical buildings. Based on multi-stakeholder work carried out as of 2011, a strategic plan was agreed upon at the institution for the preservation and enhancement of this historical architectural complex, based on the aforementioned principles, as well as others, which included: preserving the institution’s uniqueness and identity; forging closer tiers with the city, especially with the territory in which the institution is situated; ensuring the sustainable requalification of the Nahm; increasing the offer of sociocultural activities and science and health education for the population; and developing a park campus that is open to the public ( Fiocruz/COC, 2014 ; Pinheiro et al., 2019 ).

Sustainable requalification goes beyond the revitalization of historical areas and the more efficient use of natural resources to reduce environmental impacts. In its application to the heritage in question, it aims to: improve the work and life of the users of the Nahm and the Manguinhos-Maré Campus; enhance the value of the cultural heritage of the campus; improve social cohesion by promoting citizenship and valuing diversity; and foster socioeconomic revitalization for the territory. Its conception was influenced by the work of Rehabimed ( Consorsio Rehabimed, 2008 ), an interdisciplinary network focused on sustainable interventions with a view to the socioeconomic revitalization of historical city centers in the Mediterranean ( Pinheiro et al., 2019 ). Similarly, the idea of a park campus open to the public is envisaged as a fundamental strategy for strengthening the territoriality of the Nahm, enabling a broader range of activities and making the place more plural and democratic.^13^ Nonetheless, it also presents a challenge to the institution, whose core activities are academic research and science and technology production.

The plan, which actually covers the Nahm plus some of the modern buildings, has a specific methodology for ordering and prioritizing the actions and is subdivided into programs, each with its own characteristics and demands, for each thematic area deemed necessary for the success of such a major requalification endeavor.^14^ At the time of writing, it encompassed: the Architecture and Urban Program, the Exhibition Program, the Fundraising Program, the Technical Cooperation Program, the Communication Program, the Welcome Program, and the Sustainability Program.

At this point, it is worth focusing on the Sustainability Program, designed to expand and disseminate strategies for the sustainability of the long-term requalification plan, guided mainly by Agenda 2030 ( ONU Brasil, 2023 ), the Common Conceptual Framework on Sustainability, and the Institutional Program for Sustainable and Healthy Territories (Fiocruz). The program is divided into four phases, some carried out concurrently and others successively, starting with an exploratory phase involving dialogue between different actors and researchers to collectively develop concepts related to sustainable requalification. It also included a research phase, in which data were generated with the community of COC workers. Its principles and action guidelines are being consolidated and documented, as are the proposals for alignment and coordination with other programs.

Access to different parts of the Nahm by internal and external publics is organized mainly by the Fiocruz Museum of Life, which arranges and runs public visits to the circuit of cultural activities offered at the Manguinhos-Maré Campus, and the DPH/COC, which adopts heritage education strategies to reach out to society and help foster emotional ties with this cultural heritage.

## Perceptions of the territory: data from a social study

There are several techniques and methods that help achieve social participation in heritage policies, which cover two different perspectives: the collective development of proposals and the appropriation of the heritage itself. For the former, mechanisms must be created to hear and interact with society at large, including spaces to receive and integrate the contributions and experiences of each individual in their complexity and subjectivity. On the plus side, there are countless strategies that can be employed to promote permeability, fruition, use, and shared experiences in these spaces to forge emotional ties and promote values that are expressed in everyday interactions and contribute to the appreciation and preservation of the heritage.

These two strands of social participation are interrelated and influence each other, enabling deep relationships to grow between individuals and the cultural heritage. This in turn gives rise to increased interest and desire for integrated conservation and sustainability. Therefore, understanding the territory’s perceptions of the Nahm Requalification Plan is a vital step in encouraging reflections on social participation in its implementation process.

This part of the article is based on social research carried out within the scope of a master’s program ( Almeida, 2021 ), which aimed to promote critical reflections on and contribute to strategies to foster the social participation of groups from the territory in the design of integrated and sustainable propositions for the Nahm Requalification Plan. According to Antonio Carlos Gil (2008 , p.26), social research can be defined as “the process that, using scientific methodology, allows new knowledge in the field of social reality to be obtained.” Through the methodology of qualitative social research, it is possible to identify people’s perceptions, feelings, behaviors, opinions, and beliefs.

The methodology involved the administration of online questionnaires, the responses to which were collected between November 2020 and March 2021. Since social research is inextricably linked to the socioeconomic and political factors specific to its time and depends on this context to be understood, we should explain that the research was conducted online because of the covid-19 public health crisis. This profoundly affected people’s work, education, housing, food, transport, and other social conditions, both individually and collectively, impairing the health of society and exacerbating preexisting social and economic disparities. In Brazil, and especially in Rio de Janeiro, these factors had a cruel impact on people who were already in a state of socioenvironmental vulnerability, especially *favela* dwellers. Therefore, it is important to note that the groups of interest to this research were not only part of this scenario in their own specific ways, but they came from some of the hardest hit populations, making this one of the most significant factors.

The research was geared towards two groups of interest, which were selected to take account of the inseparability of the cultural heritage in question from its local territorial dynamics, embedded in a polysemic space formed of a set of forms, objects, actions, and human interactions, over which Fiocruz as a social actor exerts power. The two groups consulted were the Fiocruz community (internal) and the residents of the territory (external), understanding the importance of each one for the social participation of the Nahm.

After an exploratory survey, initial contact was made, which was follows by a link to the questionnaire. Different strategies were used to get the research out to the groups of interest, such as the snowball effect, in which each respondent nominates and forwards the questionnaire to others; a social media page and a website presenting the research; and transmission via the institution’s mailing list. The questionnaire was designed in such a way as to consider the specific features of each group, paying attention to their relationship with the institution. The following topics were covered: knowledge about the Nahm and its requalification plan; use of and routine activities in these spaces prior to the pandemic; experiences of visiting the Fiocruz Museum of Life circuit; and interest in participating collectively in developing proposals. The analyses presented in this article seek to understand the relationship of these actors with the Nahm, focusing on their perceptions of its requalification plan.

## Internal community of Fiocruz

The internal group consisted of Fiocruz employees and outsourced workers, students, researchers, and retirees from the different Fiocruz campuses, including the regional units of other states. The sampling calculation was based on an institutional document on the Fiocruz workforce containing data from 2020 ( Fiocruz, 2020 ), and the sample size achieved had a confidence level of 90% and maximum error of 5%. A total of 274 valid responses were received.

The respondents from Fiocruz’s internal community were primarily workers aged between 35 and 64, with master’s or doctoral degrees, and were from the Manguinhos-Maré Campus. As evidenced elsewhere (Almeida, Pinheiro, 2022), this group’s permeation in and appropriation of the Nahm spaces was associated largely with their regular work and study activities, which in this research is classified as a “close relationship.” Attention should be paid, however, to the capacity of fairs and events organized in the gardens and urban spaces of the complex to help deepen the relationships and values attributed to this heritage, as these were activity options indicated by the participants.

Despite demonstrating a routine relationship with the Nahm, most of the respondents from this group indicated that they did not know the requalification plan (approximately 60%), or else knew about it but were not sure what it was (23%). Among those who had heard of the plan before the survey (40%), the most effective communication strategies involved posts online and interaction with coworkers, which jointly accounted for more than 65% of this item (Almeida, Pinheiro, 2022, p.501-502).

The research included hypothesis testing using simplified methodologies, which are fundamental for understanding Fiocruz’s cultural heritage based on the principles of integrated conservation and sustainability, and the conception of the Nahm in its (inter)relationship with the actors who constitute its territory. Chief among these was the calculation of Yule’s Q ( Gil, 2008 , p.162-167), which serves to measure the degree of association between two variables. It was used in this study to analyze the degree of correlation between routines in the spaces of the Nahm and knowledge of its requalification plan. The result indicated a moderate positive correlation, which demonstrates the importance of establishing a close and routine relationship with the heritage through a diversity of uses, as this contributes to a sense of appropriation of the cultural heritage and helps the spread of knowledge about its requalification plan. This underlines how important integrating cultural heritage into social life is for promoting integrated conservation and sustainability.

The limited knowledge on the part of Fiocruz’s internal population about the Nahm Requalification Plan points to some challenges regarding their participation in the collective and integrated construction of proposals, in view of the difficulties in disseminating its basic information. Nonetheless, 72% of the respondents from this group expressed interest in such engagement. This could be linked to the institution’s identity, especially the importance of the main building (Moorish Pavilion) and the overall complex as a political, cultural, and institutional emblem, and it could also be a consequence of the participatory management approach taken by Fiocruz since the 1980s (Almeida, Pinheiro, 2022).

The respondents who indicated that they knew about the plan were asked to provide a brief description of what they understood it to be. The purpose of this question was to assess how clear the public communication messages were and how well they were being understood by the target audience. Content analysis ( Bardin, 1977 ) was used to analyze the participants’ responses, which involved their organization, coding, and categorization in separate stages.

Eighty respondents included words that represented an objective, the main ones being: “preserve,” “enhance,” “requalify,” “conserve,” “recuperate,” and “restore.” Thirty of these responses related these objectives to the use of the spaces, with the aim of rearranging, changing, or improving their uses, proposing new or varied uses, and even promoting sustainable use. Regarding the purpose of these actions and the Nahm spaces, 42 respondents pointed to scientific communication, sociocultural activities, visits, accessibility, museum, education, institutional demands, leisure, and exhibitions.

Eighteen respondents identified the public targeted in the plan using the words: “society,” “workers,” “surroundings,” “city,” “community,” “Fiocruz,” and “visitors.” Note that the word “territory” was not used by any of the respondents. This is a term that has been called for by several COC and Fiocruz working groups, mainly by the Social Cooperation Coordination, and is also used in the institution’s documents and policies; however, it has not yet been absorbed by the workers.

**Figure 5:**
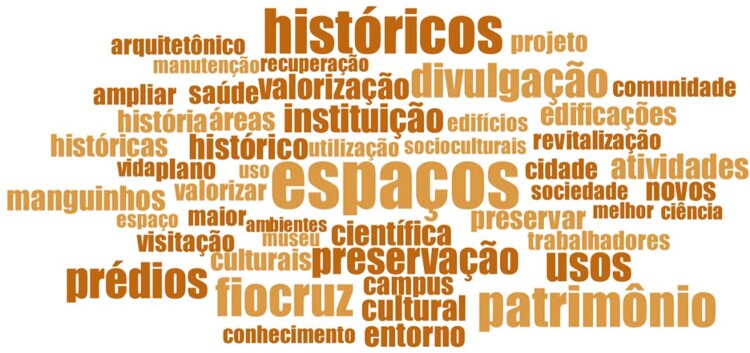
: Word cloud on understanding of the Nahm Requalification Plan by the Fiocruz internal community ( Almeida, 2021 )

In the word cloud shown in Figure 5 , font size is associated with the frequency of use by the respondents, revealing the key topics for this group in the study period.

The most prominent words used to describe the Nahm Requalification Plan were “uses” ( *usos* ), “spaces” ( *espaços* ), “historic(al)” ( *histórico* ), “heritage” ( *patrimônio* ), “cultural” ( *cultural* / *culturais* ), “valuing/to value” ( *valorização* / *valorizar* ), “communication” ( *divulgação* ), “buildings” ( *prédios* ), and “Fiocruz.” This shows the plan was understood as proposing new, mainly cultural uses, reinforcing the image of the complex as heritage of great historical value. The use of the word “Fiocruz” reveals the strength of the institutional identity among this group, and is also associated with the appreciation of the Nahm space, upon which it so heavily depends.

There was also an item on the geographic proximity of the Nahm to the participants, which was based on the following question: does the way in which the Fiocruz internal community is distributed in the Manguinhos-Maré Campus influence this community’s appropriation of the Nahm heritage and knowledge about the plan? That is, does the distance between the workplace and the Nahm directly impact the dynamics of use of its spaces and knowledge about its requalification plan? In order to do this analysis, the responses of the participants from the Manguinhos and Maré (former Expansion) campuses were analyzed according to their location. These locations – based on answers given to the questions about their place of work/study before the pandemic and the unit to which they were affiliated – were then identified on an institutional map of the campus.

**Figure 6:**
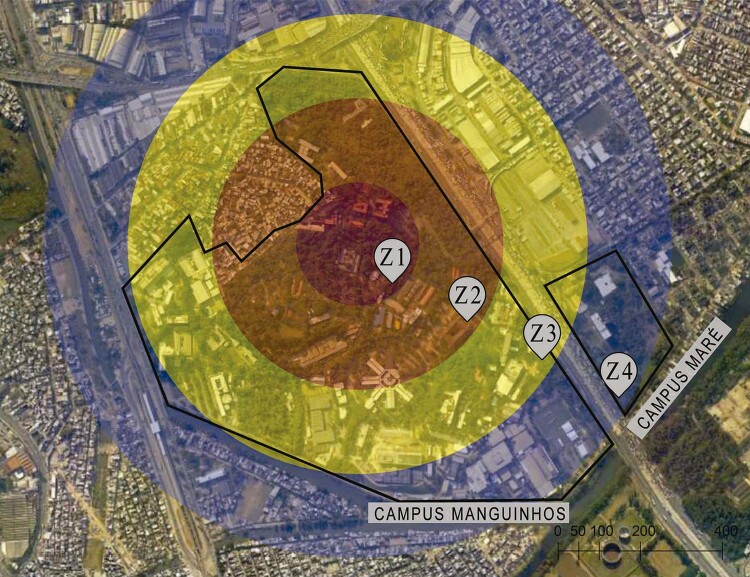
: Zoning of the Manguinhos-Maré Campus used for the analysis (adapted from Almeida, 2021 )

The territory was zoned along the same lines as heat zoning, with a central zone (zone 1, Z1), containing the Nahm, colored red. The rest of the region was then divided into three more equidistant zones (Z2 = orange, Z3 = yellow, Z4 = blue). The building of the National Institute for Quality Control in Health, which straddles two zones, was included in Z3 ( Figure 6 ).

The zone-based analysis revealed that the 243 respondents from the internal group were distributed as follows: 32 in Z1, 71 in Z2, 99 in Z3, and 41 in Z4. Starting from two categories created to represent the degree of relationship with the Nahm (“close relationship” and “distant relationship”) and associating them with the geographical position of these participants, it was possible to ascertain whether those closest to the heritage were the ones who most experienced and appropriated the spaces of this heritage complex in their daily lives.

The respondents who worked or studied in the red zone (Z1) before the pandemic were the ones who most related to the Nahm in their daily lives, with over 90% expressing a close relationship. The respondents from the orange and yellow zones (Z2 and Z3) had a slightly less close relationship with the Nahm spaces, with approximately 65% having a close relationship. Finally, just 51% of the respondents from the furthest zone (Z4) expressed a close relationship with the heritage. The data therefore confirm the hypothesis that those who worked or studied geographically further away from the historical heritage were less likely to establish a close relationship with its spaces.

Using the same zones, it was also investigated whether the respondents’ geographic location influenced their knowledge of the Nahm Requalification Plan. For this question, it was necessary to adopt one more criterion: respondents who worked or studied at COC were disregarded, as this group was found to be very likely to know about the plan, which would mask the perceptions of the other respondents. This phenomenon mainly occurred in Z2, which includes the Center for Documentation and History of Health and the offices of the Fiocruz Museum of Life, and Z4, in the Maré Campus, which, when the questionnaire was administered, housed workers and students linked to COC.

**Figure 7:**
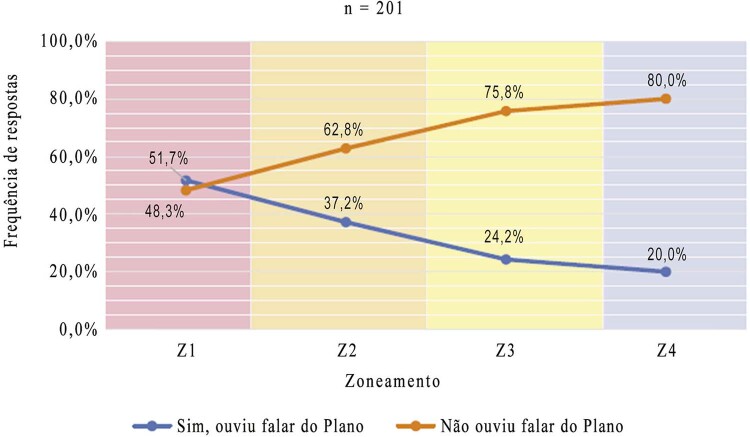
: Correlation between knowledge of the Nahm Requalification Plan and zone in the campus ( Almeida, 2021 )

Eliminating Casa de Oswaldo Cruz workers and students reduced the total for the analysis of this question to 201: 29 in Z1, 43 in Z2; 99 in Z3; and 30 in Z4. Adopting yes/no response options (“heard about the plan” vs. “not heard about the plan”) and calculating the proportion of responses per zone, it was found that the Z1 (red) was the zone with the highest proportion of respondents who knew about the plan. This was the only zone in which more respondents knew about the plan than did not. In the other zones, there were more respondents who did not know about the plan than who did, and the proportion of negative answers rose with increased distance from the Z1 ( Figure 7 ).

Therefore, based on the zoning of the Manguinhos-Maré Campus, it is possible to state that the geographical distance between the Nahm heritage and the workplace of the members of the internal community directly influenced their relationship with these cultural spaces and their knowledge of the requalification plan. The greater this distance, the less likely they were to experience and have frequent contact with these spaces and the less likely they were to know about the requalification plan.

## Residents of the territory

The choice of population for the external group was based initially on the fact that Fiocruz is situated in the Manguinhos neighborhood and also has a close relationship with Maré, if we bear in mind the concept of territory used in this study. It was also based on studies on the visiting public published in *Cadernos do Museu da Vida* between 2009 and 2015, as well as on work on the museum’s region of influence, published in 2020 ( Bevilaqua et al., 2020 ), in which it was found that residents of neighborhoods in the North Zone of Rio de Janeiro are the ones who most visit the spaces and participate in the activities offered by the Fiocruz Museum of Life.

Accordingly, the survey was targeted at residents from the Fiocruz territory and ultimately received 75 valid responses from residents of the following neighborhoods: Manguinhos, Maré, Bonsucesso, Jacaré, Jacarezinho, Higienópolis, and Benfica. A variety of strategies were adopted to communicate the research and disclose the link to the online form, including the creation of a website and profile on Instagram, and sharing messages via email, WhatsApp, and Facebook. Although the number of responses is not statistically representative of the population in the target area, the results encourage reflections on the theme of social participation in a requalification plan.

The respondents from the external group were mainly from Maré and Manguinhos, aged between 25 and 44, and graduated from high school or university. As observed by Almeida and Pinheiro (2022 , p.503), this profile may be related to the use of online research, which tends to reach younger people with higher education. However, it is worth highlighting the role of the Census as an auxiliary instrument in the analysis of populations, and the need for its data to be updated, since at the time of writing the most recent Census data on the study area were from 2010.

The relationship of the respondents from the external group with Fiocruz was developed mainly in activities offered to the public by the Fiocruz Museum of Life, events and campaigns in the area of health, and public health services provided by Fiocruz. This type of participation in institutional activities is indicative of sporadic, irregular access by this group, but which is still sufficient to promote knowledge of the Nahm, since almost all the respondents knew that the campus had historical buildings.

The main way in which the permeability and fruition of the Nahm spaces was fostered for this external public (for almost 70% of the respondents) was through the mediation of the museum and participation in events, making these the main channels for gaining familiarity with its cultural heritage (Almeida, Pinheiro, 2022). Twenty of the respondents also replied that they had been involved in some project or course at the Nahm spaces and 13 had engaged in programs offered by the Manguinhos Workshop School or the Fiocruz Museum of Life. These are inclusive experiences that promote use of and access to the heritage.

When asked if they knew that Fiocruz was carrying out a project to improve and expand public access to its cultural spaces, called the Nahm Requalification Plan, the majority (83%) indicated that they did not. This can be interpreted as indicating a dearth of strategies for publicizing the plan at the time of the research among the external public. Direct communication (word of mouth) was one of the most effective means of communication about the plan prior to the study, particularly via coworkers and neighbors (total of six). Some participants also reported that they learned about the requalification plan from being approached for the purposes of this study.

In order to comprehend the respondents’ understanding of the plan, the same strategy was used as with the respondents from Fiocruz’s internal community: an open question giving respondents the opportunity to briefly describe their understanding. This field in the questionnaire was available to those who replied they had heard about the plan and was completed by 11 respondents. The data were analyzed, coded, and grouped using collective subject discourse. This methodology understands research participants as individual speakers who represent a collective. It can be used to analyze qualitative questions with a low response rate. According to Fernando Lefevre and Ana Maria Cavalcanti Lefevre (2006, 2014), an individual is able to express the opinions shared by his/her community and how his/her social relations with the environment and with other actors influence his/her history and cultural development and therefore his/her speech.

Despite being constructed in the first person singular, the sentence yielded by this methodology represents a collective person, namely, the group of residents who had heard about the Nahm Requalification Plan. This sentence was: “I still don’t really get it. I understand that the proposal is to resignify, revitalize, and repurpose the spaces to increase public visitor numbers and social and cultural inclusion.” From this result, we can see that this group of respondents indicated that they understood the objective of the plan to enhance public participation in the Fiocruz Manguinhos-Maré Campus and to promote social and cultural inclusion, but were not clear how this would be achieved.

Although few were aware of the Nahm Requalification Plan beforehand, there was strong interest in participating in the collective construction of its proposals (Almeida, Pinheiro, 2022, p.503), either in general or in specific areas. However, the manifestation of this view did not correlate directly with the residents who had a closer relationship with the Nahm. That is, interest in participating in the plan was expressed irrespective of the respondents’ relationship with the cultural heritage. This could be explained by a broader historical relationship with Fiocruz and participation in its social projects.

## Final considerations

The results analyzed in this article demonstrate how difficult it is to effectively communicate the Nahm Requalification Plan in a way that raises awareness and informs the public of the Fiocruz territory, which is understood here as being constituted of both the workers and students of the institution and the residents of contiguous neighborhoods. They point to the importance of creating spaces for ongoing dialogue, exchange of experiences, and signification of the Nahm heritage and its plan, on the basis of the understanding that cultural heritage is inherent to social systems, which are constructed and (re)produced through an unceasing process of interaction ( Santos, 2017 , p.316). Workshops, debates, and heritage education actions could be harnessed to this end with the aim of expanding the recognition and communication of the historical heritage and its requalification plan in order to deepen socio-affective relationships and expand the participation and collaboration of all the stakeholders.

The structure of the programs for the Nahm Requalification Plan, developed by heterogeneous working groups focusing on each topic, is important for enabling informed, diversified discussions on the guidelines and orientations for the plan, representing an important step in participatory terms, bringing in different departments and professionals both from Casa de Oswaldo Cruz and other Fiocruz units. However, there is still the unresolved challenge of how to achieve the involvement and representation of the sociocultural groups from the territory external to the institution, which is so important for shared and collaborative governance – a key aspect of integrated conservation and sustainability – so that real, meaningful territorial development can be achieved, opening spaces for two-way exchanges with socioenvironmentally hidden populations.

In this study, hypothesis testing enabled associations to be identified between knowledge about the plan and intensity with which the Nahm heritage was appropriated by the groups and included in their routine activities, with increased levels of receptivity and involvement being associated with involvement in the territory and periodic experiences of the heritage. This reinforces the importance of integrating cultural heritage into social life, in line with the precepts of integrated conservation, together with the adoption of strategies to raise awareness and reach out to those who are geographically and emotionally more distant from the heritage, helping to promote social participation and consolidate the vocation of the Nahm not only as cultural heritage for science and health, but as an asset with symbolic, cognitive, cultural, and identity values and a structuring element for sustainable development.

The research on which this article is based was carried out in 2021, since which time the Nahm working groups and programs have prepared and communicated a variety of information in different online and in-person media. The resumption of in-person work activities, blended with remote working, and the reopening of the Fiocruz Museum of Life are recent factors that impact the permeability and use of the Manguinhos Campus and its historical heritage. Furthermore, the Nahm Sustainability Program has been presented as a strategy to boost social, political, cultural, environmental, and economic outreach since the Requalification Plan was rolled out. New surveys and research would therefore be worthwhile to reassess the knowledge, inclusion, and involvement of different groups from the territory in the construction of a plural and democratic park campus that reflects the vitality of the setting.

Until 2022, Manguinhos Campus was understood as being quite separate from the rest of the Fiocruz premises. Since the Expansion area became the Maré Campus and was physically connected to Manguinhos via a footbridge over Brasil Avenue, it has been possible to see the two campuses as one – the Manguinhos-Maré Campus – bringing them into line with the idea of a park campus. The concept of this type of campus is somewhat fluid and subject to different constructions in the social imaginary, but at its heart it is about being a healthy, safe, comfortable, and culturally enriching environment for its workers, students, and visitors, and, crucially, one that is open to the public. As previously mentioned, this is a challenge for the institution, as the campuses host intense academic activity and scientific and technological production. Indeed, a closer look at each of these campuses shows they have distinct characteristics that make them more or less suitable for the function of a park campus.

The Manguinhos Campus, with its larger area, significant vegetation cover, a preservation area with historical buildings and gardens, and a consolidated trajectory of receiving the public, is a more natural candidate for a park campus. The Maré Campus, for its part, with its smaller dimensions and occupation patterns, has more buildings and hosts activities that demand higher levels of biosafety, making it more challenging to think of it as a park campus. Nonetheless, it cannot fail to be seen as a strategic agent for sustainable territorial development and a greater appreciation of local memories and identities not only in Maré, but across the entire region, provided it is treated as part of the unified Manguinhos-Maré Campus. Envisaged thus, let us hope that, grounded in a sustainable requalification plan for their historical areas, the whole campus can bring about a dialogical relationship between cultural heritage, territory, and social participation.

## NOTES

1 In *História de pessoas e lugares* , Tania Maria Fernandes and Renato Gama-Rosa Costa (2009) indicate proximity to what was then the Oswaldo Cruz Institute as one factor behind the occupation of Morro do Amorim on account of employment opportunities at the institution. It is no coincidence that this *favela* is now also known as Oswaldo Cruz Park, underlining its relationship with Fiocruz.

2 According to a city ordinance (Brasil Avenue gate [1954-1955]) (Oliveira, Costa, Pessoa, 2003), the following buildings were erected from the 1940s onwards: the Carlos Chagas Pavilion (1944), Arthur Neiva Pavilion (1947-1951), the Central Dining Hall Pavilion (1948-1953), the Biology Pavilion (1947), and the Henrique Aragão Pavilion (1954-1960).

3 A Fiocruz technical-scientific unit created in 1986 to produce and spread historical knowledge on health and the biomedical sciences, to preserve and enhance health heritage, and to educate in its spheres of action and engage in science and technology communication activities related to health.

4 DPH/COC is one of the four departments of Casa de Oswaldo Cruz and is dedicated to preserving and promoting Fiocruz’s architectural, urban, and archaeological heritage.

5 In 1980, case #1.037-T/80 was opened by Iphan in response to a Fiocruz request, which resulted in the listing at the federal level, on January 29, 1981, of the Moorish Pavilion, the Clock Pavilion, and the Stables. In 1986, also at Fiocruz’s request, a new process was started at Iphan (#40099.060054/86-41; not yet concluded) to extend the federal listing to include other buildings from the eclectic period (Evandro Chagas Hospital, Figueiredo de Vasconcelos Pavilion, Dovecot, and Yellow House), as well as an area called the preservation area of the campus and assumed to be listed, as set forth in an injunction of a type permitted under Brazilian legislation. This area also contains the modern buildings, two of which (Arthur Neiva Pavilion and Central Refectory) were listed in 2001 by the State Institute of Cultural Heritage (Instituto Estadual de Patrimônio Cultural, Inepac).

6 The master plans in force at the time were the 1988 Manguinhos Campus Master Plan, developed by the institution under the technical coordination of the Brazilian Institute of Administration (Instituto Brasileiro de Administração, Ibam), and the Oswaldo Cruz Foundation Campus Environmental Master Plan, from 1996, developed by the landscape designer Fernando Chacel.

7 For the development of Occupation Plan, a management committee was set up under the coordination of DPH/COC with members from COC and other Fiocruz units, the President’s Office, and heritage protection agencies (e.g., Iphan and Inepac), with Ibam as a consultant. The Occupation Plan was approved by Fiocruz’s Deliberative Council in December 2013 and, in addition to later inspiring a master plan for the entire campus and the Nahm Requalification Plan, it became a central part of the transformation of this campus into an Area of Special Functional Interest by the Rio de Janeiro municipal authority.

8 Of note, the democratic governance approach adopted by Fiocruz to guide the projects and work processes of its specific bodies. Its organizational structure includes collegiate bodies that help ensure management and deliberation is shared with the institution’s employees.

9 Sustainability was originally conceived as being constituted of environmental, social, and economic aspects. However, some studies have suggested including other dimensions, such as the 2019 Common Conceptual Framework for Sustainability of Ibero-American Museum Institutions and Processes, which includes the cultural dimension, and the study by Ignacy Sachs (2004) , which considers eight integrated dimensions, including the national and international political dimension.

10 The 2030 Agenda was approved by the UN General Assembly in 2015, establishing 17 Sustainable Development Goals with a view to eradicating poverty and promoting economic, social, and environmental development on a global scale. SDG 11 comprises ten targets to be achieved by 2030 and deals with sustainable cities and communities (see ONU Brasil, 2023 ).

11 “Tokenism” derives from token, or symbol, and references an article by Martin Luther King published in 1962 in which he criticizes the use of symbols to create the illusion of greater integration. It is applied to give a sense of greater participation and inclusiveness for hidden social groups and minorities based on symbolic, but ultimately minimal and insignificant representation (Radi, 30 jul. 2019).

12 Social participation in policymaking has been shown to be a challenge and a fundamental principle for the implementation of these policies and their effective social appropriation. This premise applies to the policies of both governments and public institutions. The challenge of this form of social participation can be analyzed from examples like that of Fiocruz, which has devoted itself to building institutional policies in its spheres of influence in a participatory way, involving different institutional actors and inviting evaluation by external specialists and its workforce. In the area of cultural heritage, its policy for the preservation of scientific and cultural collections, produced in 2018 and updated in 2020, is a notable case in point ( Fiocruz/COC, 2020 ).

13 The concept of place is linked to the concept of space, but it is expanded by also taking account of the socio-affective relationships that are manifested and the values attributed. According to Yi-Fu Tuan (1983 , p.12; published originally in 1930), “in experience, the meaning of space often merges with that of place. ‘Space’ is more abstract than ‘place’. What begins as undifferentiated space becomes place as we get to know it and endow it with value.”

14 The Nahm Executive Working Group embodies the participatory governance structure. It has representatives from multiple areas of Casa de Oswaldo Cruz and promotes dialogue and integration around different proposals. This arrangement is propitious for the autonomy of the work processes. It encourages qualified and participatory debate and helps ensure the feasibility of the plan’s proposals through integrated strategic planning.
